# Supplementary material for: Epoxide-functionalization of polyethyleneimine for synthesis of stable carbon dioxide adsorbent in temperature swing adsorption
Source: Nat Commun. 2016 Aug 30;7:12640. doi: 10.1038/ncomms12640 (PMC5013602; doi:10.1038/ncomms12640)
Supplement: Supplementary Information — Supplementary Figures 1-13, Supplementary Tables 1-3, Supplementary Notes 1-3 and Supplementary Reference [file ncomms12640-s1.pdf]

Supplementary Figures

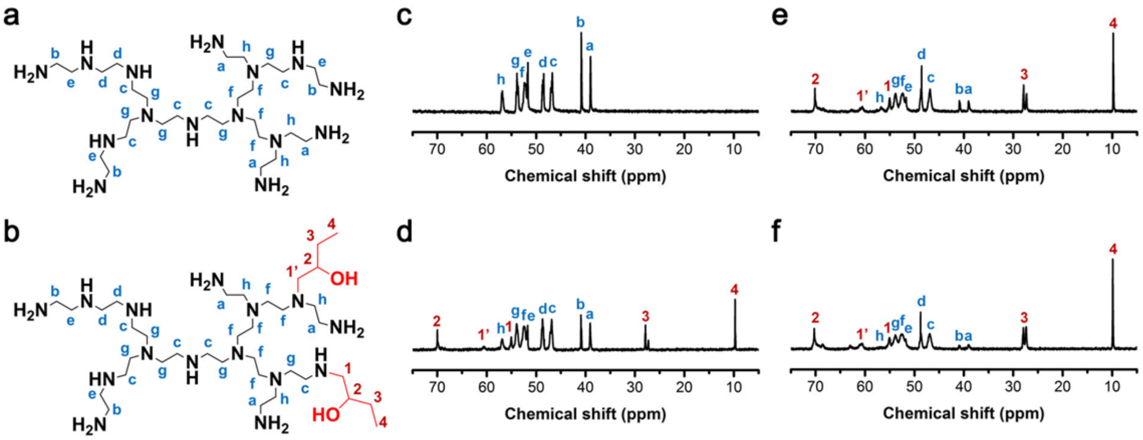

Supplementary Figure 1 | Molecular structure and ^{13}C NMR spectra for the PEI and functionalized PEIs (*n*EB-PEI). a-b, Molecular structure of PEI (**a**) and functionalized PEIs (*n*EB-PEI) (**b**). **c-f**, Liquid-phase ^{13}C NMR spectra for PEI (**c**), 0.15EB-PEI (**d**), 0.37EB-PEI (**e**), and 0.54EB-PEI (**f**).

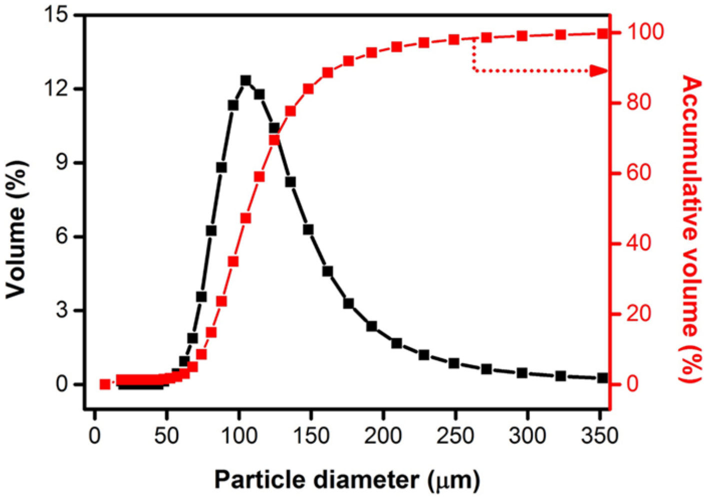

Supplementary Figure 2 | Particle size distribution of the silica spheres. The particle size distribution was measured using Microtrac Bluewave particle size analyser.

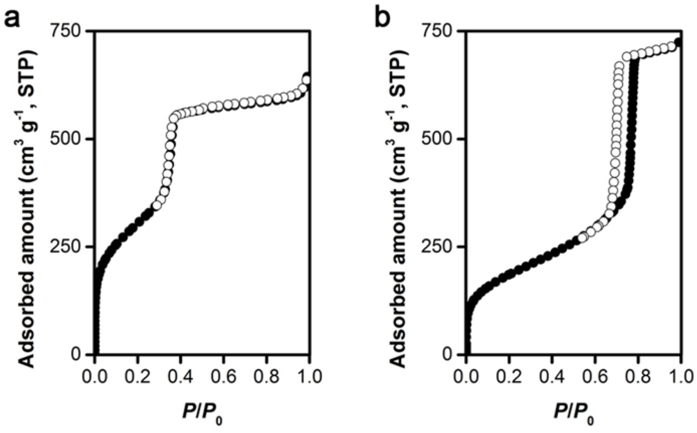

Supplementary Figure 3 | N_2 adsorption-desorption isotherms of MCM-41 and SBA-15. a-b, N_2 adsorption-desorption isotherms of MCM-41 (**a**) and SBA-15 (**b**) measured at -196 ˚C.

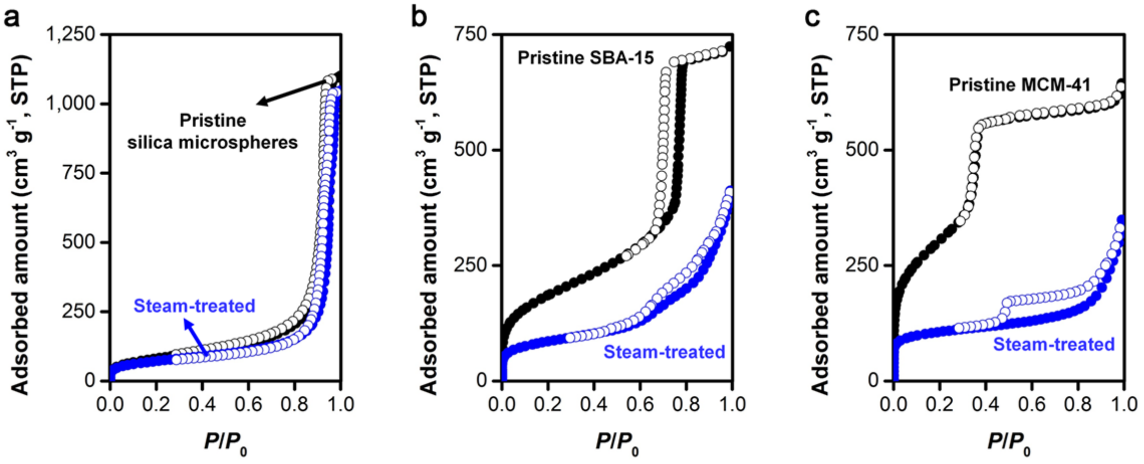

Supplementary Figure 4 | Steam-stability of PEI/silica adsorbents. a-c, N_2 adsorption-desorption isotherms of PEI-impregnated silica microspheres (PEI/SiO_2) (**a**), PEI/SBA-15 (**b**), and PEI/MCM-41 (**c**) after treatment under 100% steam at 120 ˚C for 7 days, followed by calcination at 600 ˚C.

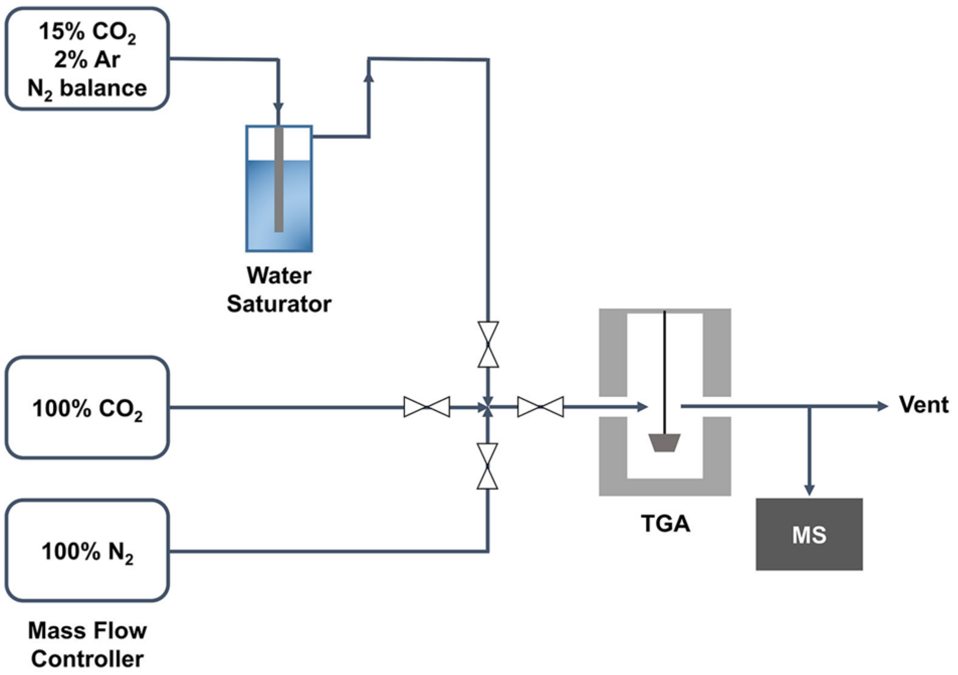

Supplementary Figure 5 | Schematic diagram of TGA-MS system. The thermogravimetric analyser (Scinco, TGA N1500) was combined with the mass spectrometer (Pfeiffer Vacuum, Omnistar GSD 320). The temperature of a water saturator was maintained at 25 ˚C.

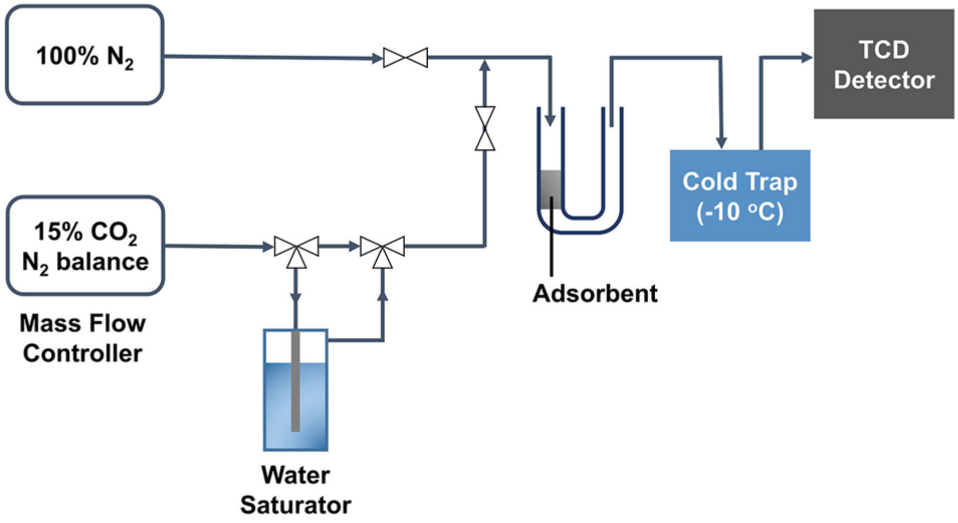

Supplementary Figure 6 | Schematic diagram of the breakthrough experimental setup. CO_2 uptake was analysed with an automated chemisorption analyser (Micromeritics, Autochem II 2920) specially equipped with a cold trap (-10 ˚C) for H_2O removal in front of a thermal conductivity detector (TCD). The temperature of a water saturator was maintained at 25 ˚C.

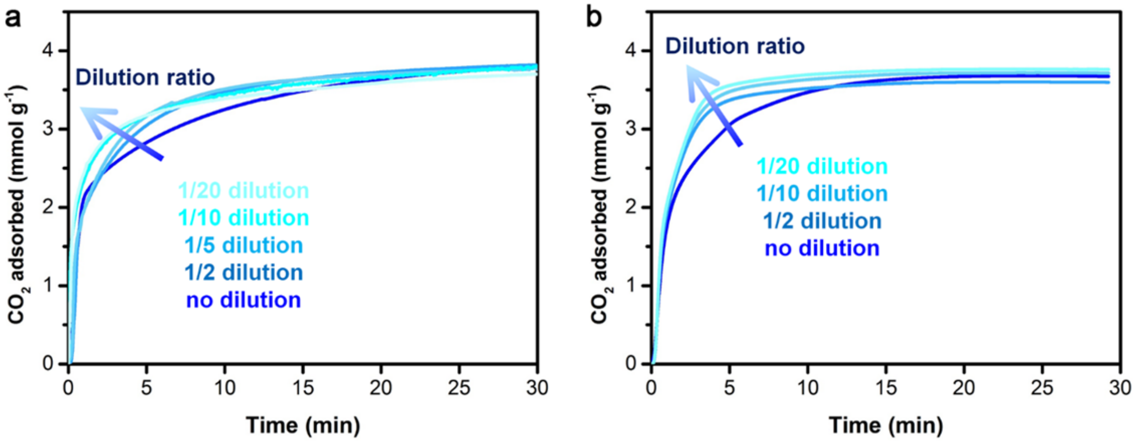

Supplementary Figure 7 | CO_2 adsorption profiles of the PEI/SiO_2 after dilution with sand in different ratios. a-b, CO_2 adsorption profiles in TGA-MS (**a**) and breakthrough setup (**b**). The results showed that CO_2 adsorption kinetics becomes substantially enhanced in both systems, as the adsorbent is diluted with more sand. Above 10-fold dilution, however, further enhancement was only marginal, which means that this dilution ratio is sufficient for avoiding the heat transfer limitation. Based on these data, we measured all the CO_2 adsorption-desorption profiles after 10-fold dilution of the adsorbents with sand afterward.

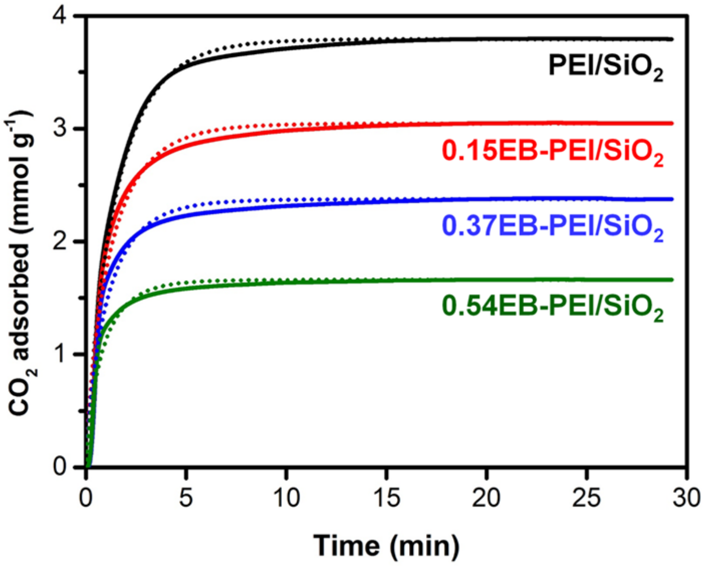

Supplementary Figure 8 | CO_2 adsorption profiles of the adsorbents measured with the breakthrough experimental setup. CO_2 adsorption was carried out in a wet flue gas containing 15% CO_2, 3% H_2O and N_2 balance at 40 ˚C. The solid lines indicate the CO_2 uptake profiles of the adsorbents measured experimentally, and the dotted lines indicate the fitting curves obtained with the Avrami kinetic equation.

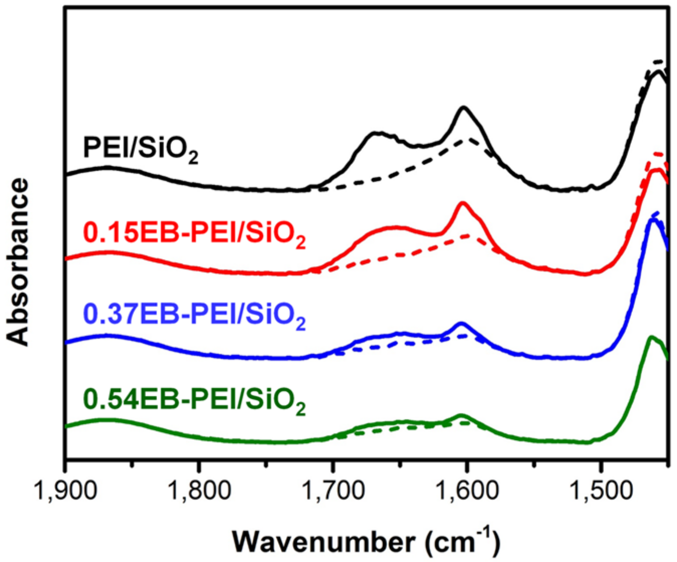

Supplementary Figure 9 | FT-IR spectra of the adsorbents after pre-treatment in a synthetic air. FT-IR spectra of the adsorbents which were measured after pre-treatment in a synthetic air (20% O_2 in N_2 balance) at 120 ˚C for 24 h in an in-situ IR cell. Dashed lines indicate the spectra for freshly prepared samples, while solid lines indicate those for pre-treated samples.

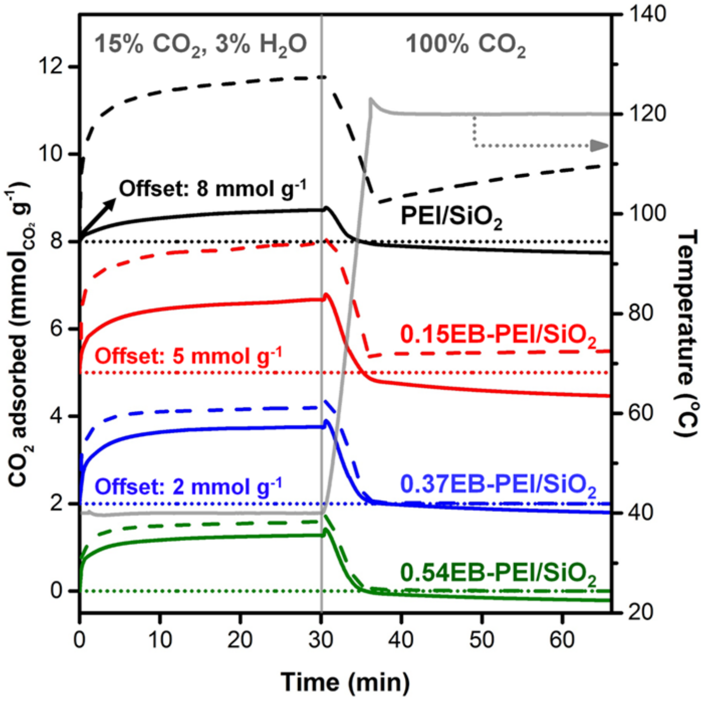

Supplementary Figure 10 | CO_2 adsorption-desorption profiles of the adsorbents after pre-treatment in dry air. CO_2 adsorption-desorption profiles of adsorbents (adsorption: 15% CO_2, 3% H_2O, 2% Ar in N_2 balance at 40 ˚C; desorption: 100% CO_2 at 120 ˚C) were measured after pre-treatment in dry air (20% O_2) for 24 h. Dashed lines indicate the profiles for fresh samples, while solid lines indicate those for pre-treated samples. The profiles for 0.37EB-PEI/SiO_2, 0.15EB-PEI/SiO_2, and PEI/SiO_2 were offset by 2, 5, and 8 mmol g^{-1}, respectively.

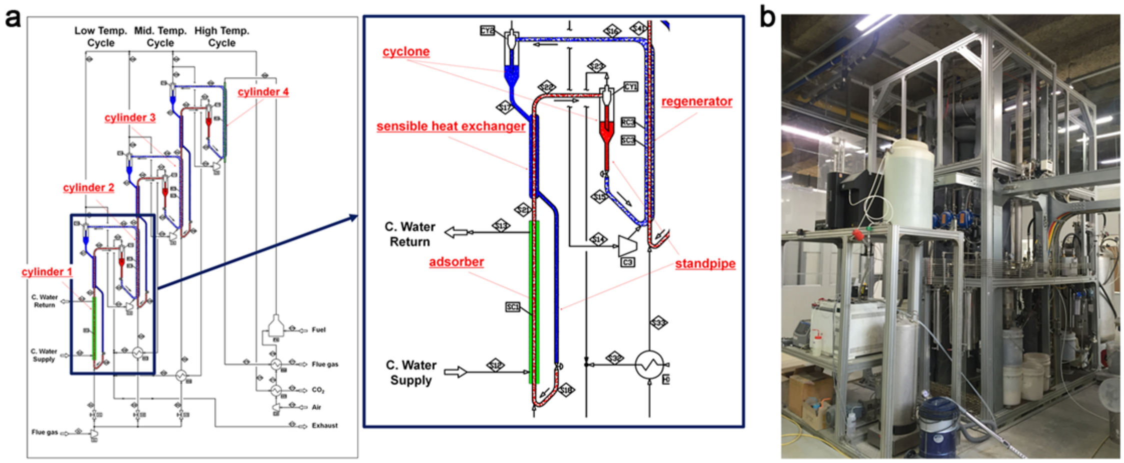

Supplementary Figure 11 | Fluidized bed setup used for test operation in a bench-scale. a-b, Schematic (**a**), and photograph (**b**) of the fluidized bed.

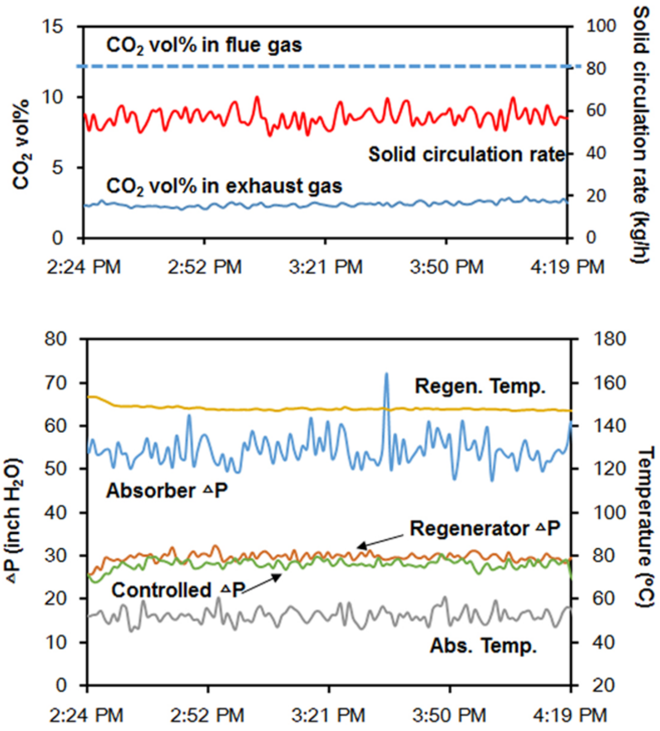

Supplementary Figure 12 | Bench-scale CO_2 adsorption experiments. The experiments were carried out in the low-temperature stage of the 3-stage fluidized bed facility described in Supplementary Figure 11. The results indicated a successful and steady-state fluidized operation of the adsorbent.

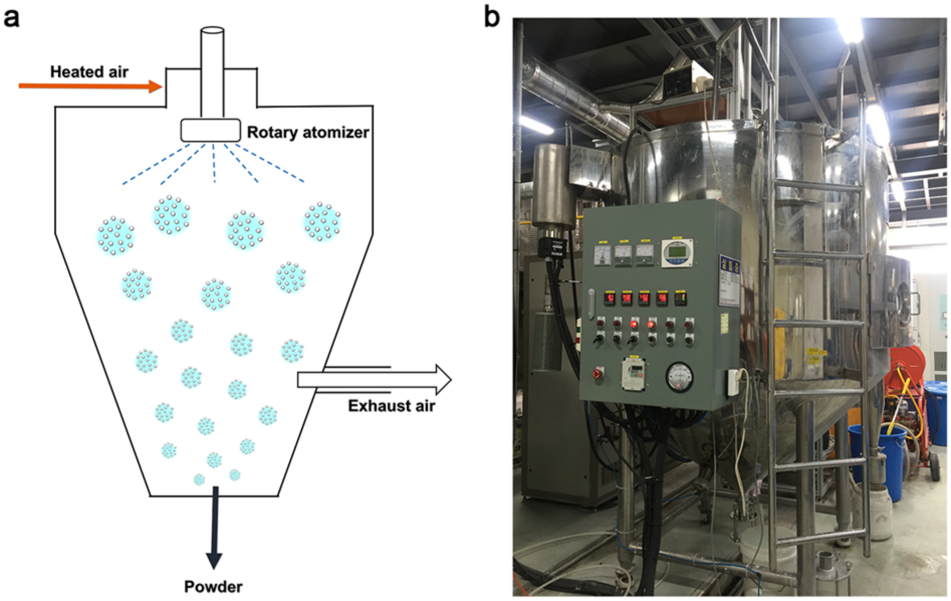

Supplementary Figure 13 | Spray dryer setup used for silica synthesis. a-b, Schematic (**a**), and photograph (**b**) of the spray dryer with a co-current drying configuration and a rotary atomizer.

Supplementary Tables

Supplementary Table 1 | Comparison of the CO_2 uptake amounts measured with two different experimental setups.

	**PEI/SiO_2**	**0.15EB-PEI/SiO_2**	**0.37EB-PEI/SiO_2**	**0.54EB-PEI/SiO_2**
**TGA-MS CO_2 adsorbed (mmol g^{-1})**	3.8	3.0	2.2	1.6
**Breakthrough CO_2 adsorbed (mmol g^{-1})**	3.7	3.2	2.2	1.8

Supplementary Table 2 | Kinetic fitting results of the CO_2 adsorption profiles (measured with a breakthrough setup) using Avrami equation.

parameters	PEI/SiO_2	$0.15EB\text{-}PEI/SiO_2$	$0.37EB\text{-}PEI/SiO_2$	$0.54EB\text{-}PEI/SiO_2$
k_a	0.67	0.81	0.92	1.2
n_a	0.88	0.83	0.82	0.82
R^2	0.9876	0.9705	0.9530	0.9507

Supplementary Table 3 | Stabilized operating condition in the fluidized bed facility.

Parameters	Values
Feed gas volume flow (L min^{-1})	175
Sweep gas volume flow (L min^{-1})	15
Solid circulation rate (kg h^{-1})	55
Feed gas mole fraction	CO_2 (0.120), H_2O (0.030), O_2 (0.187), N_2 (0.663)
Sweep gas mole fraction	CO_2 (1)
Adsorption temperature (°C)	50
Desorption temperature (°C)	150

Supplementary Notes

Supplementary Note 1: Quantitative analysis of amine state distributions in the PEI and *n*EB-PEIs were carried out using the equations shown below.

For PEI,

$$\text{Primary}(1^{o})\text{: Secondary}(2^{o})\text{: Tertiary}(3^{o}) = (A_{a}+A_{b})\text{: }(A_{c}+A_{d}+A_{e})/2\text{: }(A_{f}+A_{g}+A_{h})/3 \quad (1)$$

For *n*EB-PEI,

$$\text{Primary}(1^{o})\text{: Secondary}(2^{o})\text{: Tertiary}(3^{o}) = (A_{a}+A_{b})\text{: }(A_{c}+A_{d}+A_{e}+A_{1})/2\text{: }(A_{f}+A_{g}+A_{h}+A_{1'})/3 \quad (2)$$

where, A_i is the integrated peak area for *i* carbon species.

Supplementary Note 2: The attrition behavior of the organic-free silica microsphere and 0.37EB-PEI/SiO_2 were measured using a three-hole airjet attrition tester configured following ASTM D5757-95. 50 g of adsorbent were used for each test. As specified in the ASTM method, the test was carried out under 10 L min^{-1} air flow and the weight loss of fines was recorded after 5 h time-on-stream. The percentage of fines of pure silica microspheres collected in a thimble was 74%, while that of 0.37EB-PEI/SiO_2 was only 2.5%. The results clearly showed that the impregnation of organic polymer dramatically increased the mechanical stability of the silica microspheres.

Supplementary Note 3: The fluidized bed system is composed of 3 sets of carbonator and regenerator (Supplementary Figure 11). The facility was specially designed to use the adsorption energy released in a one carbonator for the regeneration of adsorbents in the next regenerator. It was originally designed to combine three kinds of adsorbents whose adsorption and regeneration temperatures are different. That system was already disclosed at GHGT 12 conference in 2014[1]. All the adsorbers and regenerators are riser-type. Adsorbents are introduced from the bottom and fluidized by flue gases and sweeping gases. The actual facility is made of four 4 m-tall core-shell type cylinders with 6 cyclones. The volume of the core of a cylinder is 15.5 L, and the volume of the shell is 3.4 L. The core of the first cylinder is the adsorber of the low low-temperature stage. The shell of the first cylinder is where cooling water flows to remove the heat of adsorption from the low low-temperature adsorbents. The shell of the second cylinder is the desorber of the low low-temperature stage, and its core is the adsorber of the medium medium-temperature stage. The shell of the third cylinder is the desorber of the medium medium-temperature stage, and the core of the third cylinder is the adsorber of the high high-temperature stage. The shell of the fourth cylinder is the adsorber of the high- temperature stage, and an electric heater is in the core of the fourth cylinder. The regeneration energy is supplied only to the regenerator of the high high-temperature stage, and the regeneration energy for the medium- temperature stage is supplied from the adsorption energy of the high temperature stage. The regeneration heat of the low low-temperature stage is supplied from the medium medium-temperature stage.

Our adsorbents were temporarily tested in the low-temperature stage of this facility (KCRC is currently building a new bench-scale fluidized bed facility which will be fully dedicated to the long-term test of the present adsorbents). The energy needed to run the process was supplied from the electric heater by heat transfer between spherical zeolite particles circulating in the medium-temperature stage and the high-temperature stage. A simulated flue gas containing 12% CO_2 and 3% H_2O was fed into the adsorber from the bottom of the adsorber blowing the adsorbents entering the adsorber from the connected standpipe. The solid circulation rate was controlled by regulating the pressure difference of the standpipe of the adsorber. After being separated from the CO_2-depleted flue gas by a cyclone, the carbonated adsorbents were accumulated in the standpipe of the regenerator and fed to the regenerator with sweep gas. After being separated from the product gas by a cyclone, the adsorbents were accumulated in the standpipe of the adsorber and fed to the adsorber again. Table S3 demonstrates the stabilized operating condition of the facility, and Supplementary Figure 12 shows results for the stabilized operation of the facility for 2 h. The adsorption temperature was measured at the end of the adsorber, and the desorption temperature was measured at the end of the regenerator. About 80% of the CO_2 from the simulated flue gas was captured. The pressure differences and the temperatures of the adsorber and the regenerator were maintained constantly.

Supplementary References

1. Park, Y. K. *et al.* Energy recoverable multi-stage dry sorbent CO_2 capture process. *Energy Procedia* **63,** 2266–2279 (2014).
